# Long non-coding RNAs in ferroptosis, pyroptosis and necroptosis: from functions to clinical implications in cancer therapy

**DOI:** 10.3389/fonc.2024.1437698

**Published:** 2024-08-29

**Authors:** Ke Huang, Li Yu, Dingci Lu, Ziyi Zhu, Min Shu, Zhaowu Ma

**Affiliations:** School of Basic Medicine, Yangtze University, Health Science Center, Yangtze University, Jingzhou, Hubei, China

**Keywords:** cancers, cancer therapy, long non-coding RNA (IncRNA), ferroptosis, pyroptosis, necroptosis

## Abstract

As global population ageing accelerates, cancer emerges as a predominant cause of mortality. Long non-coding RNAs (lncRNAs) play crucial roles in cancer cell growth and death, given their involvement in regulating downstream gene expression levels and numerous cellular processes. Cell death, especially non-apoptotic regulated cell death (RCD), such as ferroptosis, pyroptosis and necroptosis, significantly impacts cancer proliferation, invasion and metastasis. Understanding the interplay between lncRNAs and the diverse forms of cell death in cancer is imperative. Modulating lncRNA expression can regulate cancer onset and progression, offering promising therapeutic avenues. This review discusses the mechanisms by which lncRNAs modulate non-apoptotic RCDs in cancer, highlighting their potential as biomarkers for various cancer types. Elucidating the role of lncRNAs in cell death pathways provides valuable insights for personalised cancer interventions.

## 1 Introduction

Cell death can be classified into two groups based on its rate and susceptibility to influence by drugs or genes: accidental cell death and regulated cell death (RCD) (1, 2). Accidental cell death arises from biological processes, whereas RCD is orchestrated by signalling pathways and underlying mechanisms, maintaining homeostasis and influencing the development of various diseases. RCD encompasses apoptotic and non-apoptotic subsets, each with unique signalling induction and molecular regulatory characteristics, as well as implications for disease (3). Currently, non-apoptotic RCDs, including ferroptosis, pyroptosis and necroptosis, plays pivotal roles in cancer progression (4). Ferroptosis, an iron-dependent type of cell death, is characterised by necrotic changes in cells such as cell swelling and plasma membrane rupture, stemming from lipid hydroperoxide accumulation (5). Ferroptosis is widely acknowledged as a critical process influencing the development and advancement of various cancers (6). Pyroptosis, an inflammatory form of cell death, involves cell swelling, lysis, release of pro-inflammatory mediators, ATP production and expression of high mobility group box 1, among other features (7). Necroptosis, another form of cell death, shares morphological similarities with necrosis, such as translucent cytoplasm and organelle swelling, which can be triggered by various mechanisms (8). Cancer is a complex disease that involves the dysregulation of cell death. In cancer initiation and progression, cell death is regulated by various molecules, including long non-coding RNAs (lncRNAs).

LncRNAs, ranging from 200 to 10,000 nucleotides, lack a full open reading frame (ORF) and seldom produce small functional peptides. They are typically found in the nucleus or cytoplasm (9). LncRNAs participate in essential physiological processes such as cell cycle, tissue differentiation, metabolism and immunity (10–12). Their abnormal expression or dysfunction is frequently associated with various human diseases, including cancer (13). Previous studies have demonstrated that lncRNAs possess tissue-specific, cell-type-specific and cell developmental stage-specific properties (14–17). Moreover, the dysregulation of lncRNAs has been demonstrated to be associated with various cancer-related phenotypes, including malignancy proliferation, epithelial-mesenchymal transition (EMT), invasion and metastasis. Furthermore, lncRNAs modulate cancer progression through influencing various non-apoptotic RCDs signalling pathways.

Recent scholarly reviews have highlighted that abnormally expressed ncRNAs exert a essential role on RCDs in the progression of multiple diseases (18–20). Although the integrated roles and mechanisms of lncRNAs in non-apoptotic RCDs remain understudied, an increasing number of studies have demonstrated that lncRNAs can mediate intracellular signalling pathways, subsequently influencing physiological and pathological processes such as cancer progression (21). Herein, this comprehensive review elucidates the diverse functions and underlying molecular mechanisms of lncRNAs in modulating non-apoptotic RCDs processes during the initiation and progression of various cancers. Moreover, we also highlights the potential of these lncRNAs in cancer diagnosis and therapeutics, which have great significance for translational medicine and clinical practice.

## 2 LncRNAs in cancers

**Figure 1:**
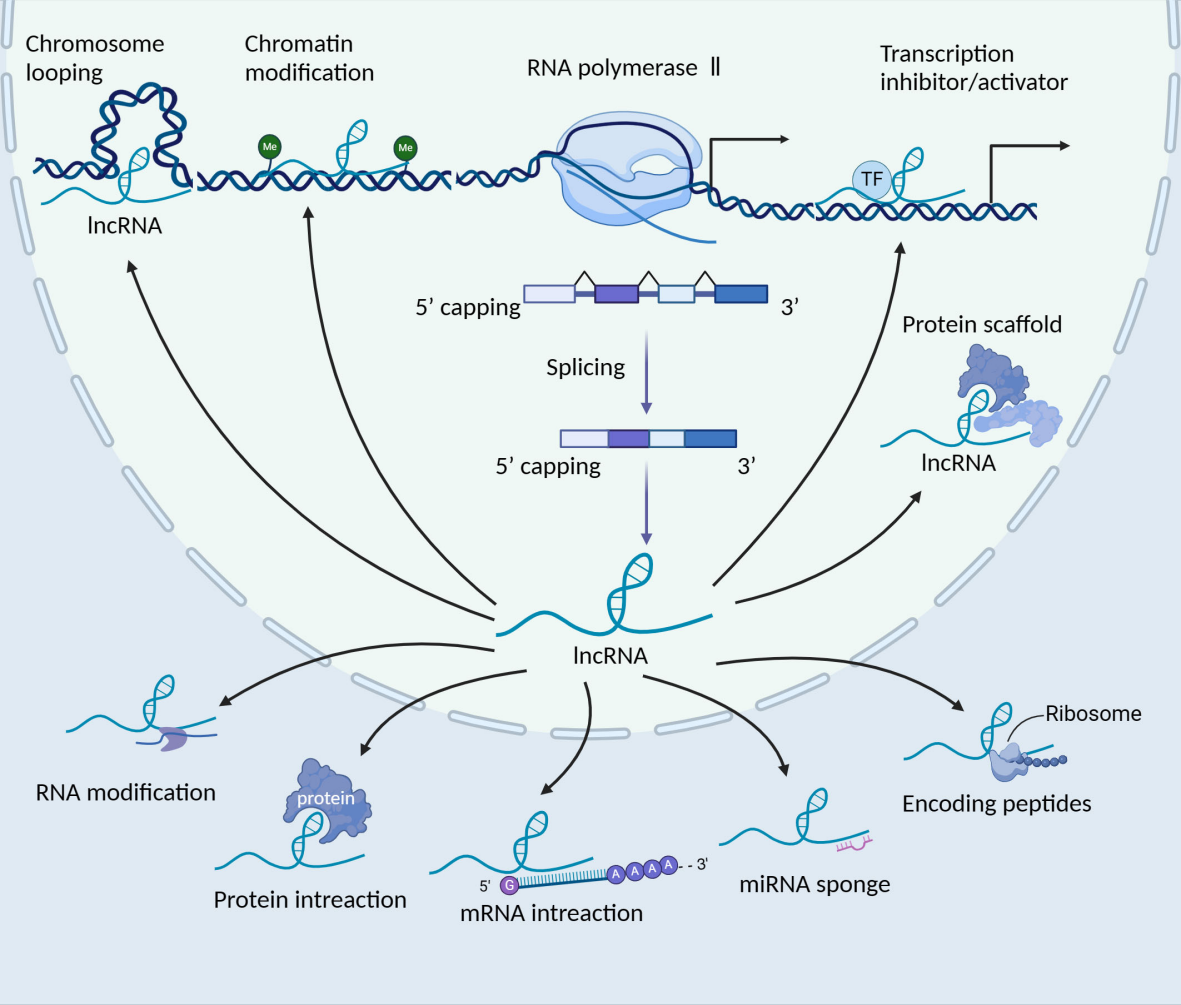
The biogenesis and function of long non-coding RNAs (lncRNAs). LncRNAs are transcribed by RNA polymerase II, localising in the nucleus or cytoplasm. In the nucleus, lncRNAs play various roles, such as the regulation of gene expression in cis or trans, splicing regulation and the formation of subnuclear domains. Certain lncRNAs, which possess 3’ cleavage and polyadenylation features resembling the consensus sequence of mRNAs, can be transported to the cytoplasm. In the cytoplasm, these lncRNAs serve various functions, such as acting as sponges for miRNAs, interacting with signalling proteins, modulating the translation of specific mRNAs and even encoding peptides. TF, transcription factor; lncRNA, long non-coding RNA.

LncRNAs are transcribed molecules longer than 200 nucleotides, which can be located in either the same or opposite direction to protein-coding genes, or within regions between genes (22). They play crucial roles in gene regulation, including the modulation of gene activation and silencing. LncRNAs perform diverse functions, including transcriptional regulation in cis or trans, regulation of gene expression and modulation of proteins or RNA molecules (23). Their mechanisms of action include chromosome looping, chromatin modification, transcription inhibitor/activator, miRNA sponging, protein interaction and translation modulation (**Figure 1**). While the majority of lncRNAs are localised in the nucleus, some also function in the cytoplasm (24). LncRNAs are transcribed by RNA polymerase II, and some of these transcripts are often polyadenylated or 7-methylguanosine capped and spliced (25–27). Typically, lncRNA functions are divided into *cis* and *trans*, including the target is near the genomic location of the lncRNA or is located on other chromosomal loci (28, 29). Within the nucleus, lncRNAs mainly regulate gene expression through interactions with transcription factors (TFs), resulting in various epigenetic chromatin modifications or DNA architecture alterations (chromosome looping) (30). In the cytoplasm, lncRNAs predominantly govern gene expression post-transcriptionally, coordinating various RNA or protein modifications to influence their activation and stability (31–33). Moreover, they can impact the stability and translation of mRNA, as well as mRNA decay, through the formation of regions with complementary sequences to mRNA (34, 35). Additionally, lncRNAs function as ‘sponges’ or ‘decoys’, competing with other genes for miRNA binding and consequently diminishing the regulatory impact of miRNAs on targeted mRNAs (36). Moreover, recent studies suggest that the translation of lncRNAs is initiated by ORFs, with certain lncRNAs carrying out their functions through their coding peptides (37). Furthermore, the dysregulation of lncRNAs plays multifaceted roles in diseases, including cancer.

Cancer is a complex disease characterised by genetic mutations, epigenetic alterations, chromosomal translocations, deletions and amplification (38, 39). In addition to mutations or abnormal expression in protein-coding genes, mutations and dysregulation of non-coding RNAs, specifically lncRNAs, are increasingly recognised for their pivotal roles in cancer (40). LncRNAs drive the acquisition of hallmark cancer characteristics, including proliferation, survival, metabolism modulation and interactions with the tumour microenvironment (TME). Recent studies have demonstrated the roles of various lncRNAs in the TME, which is a complex ecosystem involving diverse immune cells, cancer-associated fibroblasts (CAFs), endothelial cells, and the extracellular matrix (ECM), among others (41–44). It have illustrated that the roles of lncRNAs within the TME which is involved in diverse molecular mechanisms including interaction with DNAs, RNAs and proteins. Early indications of lncRNA involvement in cancer came from their transcriptional regulation by prominent oncogenic or tumour-suppressive TFs such as p53, MYC and various signalling pathways, shaping oncogenic or tumour-suppressive responses (45). Previous research has highlighted lncRNA’s participation in multiple signalling pathways, such as Wnt/β-catenin, TGF-β/Smad, STAT3 and VEGFA/VEGFR2, consequently influencing tumour invasion and metastasis, tumour angiogenesis (46–49). While the precise mechanisms through which lncRNAs participate in tumour pathogenesis remain elusive, mounting evidence suggests their key regulatory role in ferroptosis, pyroptosis and necroptosis (50, 51). Consequently, dysregulation of specific lncRNAs may exhibit anti-tumour or pro-carcinogenic functions, impacting various forms of cancer cell death.

## 3 Ferroptosis, pyroptosis and necroptosis

**Figure 2:**
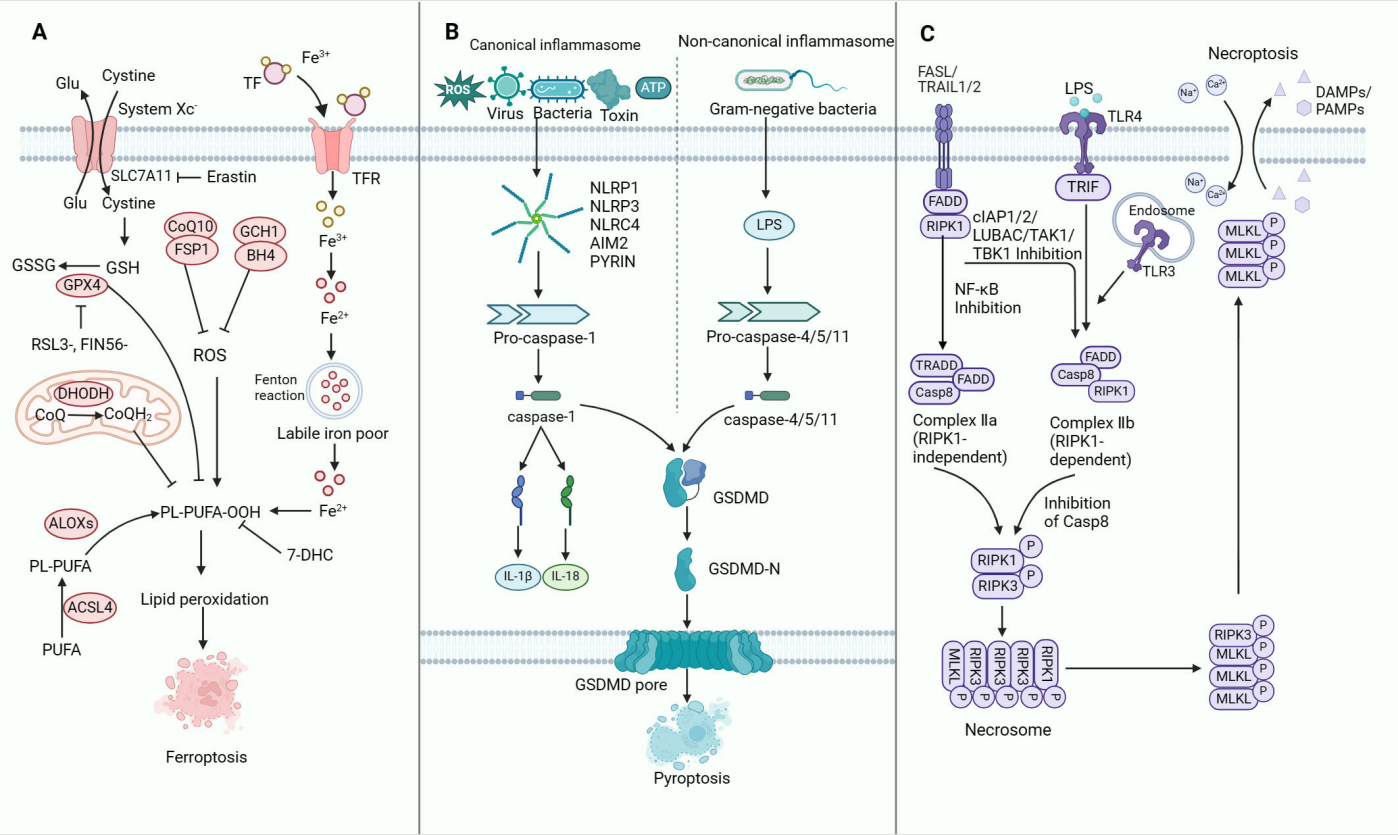
The molecular mechanisms of ferroptosis, pyroptosis and necroptosis. **(A)** Mechanisms of ferroptosis. The synthesis of PUFA, production of intracellular ROS, upregulation of iron levels and inhibition of the GPX4-dependent defence system collectively promote ferroptosis in tumour cells. **(B)** Mechanisms of pyroptosis. Upon encountering PAMPs or DAMPs, activated CASP1 or CASP11 triggers the cleavage and generation of GSDMD-N. This process plays a central role in driving pyroptosis through the activation of both canonical and non-canonical inflammasomes. The induction of pyroptosis is further modulated by signalling pathways involving ROS as well as calcium and potassium efflux. Inhibition of pyroptosis is mediated by GPX4, which counteracts ROS-mediated pyroptosis. Notably, the gasdermin-N pore-forming domains and the gasdermin-C repressor domains are distinctly separated. The gasdermin-N pore-forming domains subsequently undergo oligomerisation to form pores within the cell membrane, resulting in the disruption of membrane integrity and the initiation of cell pyroptosis. **(C)** Mechanisms of necroptosis. Activation of the death receptor results in the activation of RIPK1 and recruitment of intracellular RIPK3 to form a complex called the necrosome. Subsequently, RIPK3 is activated. Activated RIPK3 then phosphorylates MLKL, which is co-trafficked with tight junction proteins to the cell periphery, where it binds to the plasma membrane. The binding of MLKL at the plasma membrane triggers cell necroptosis. TFR, transferrin receptor; ROS, reactive oxygen species; PUFA, polyunsaturated fatty acid; GSDMD, gasdermin D; LPS, lipopolysaccharide; TLR, toll-like receptor; DAMPs, damage-associated molecular patterns; PAMPs, pathogen-associated molecular patterns; RIPK, receptor-interacting serine-threonine kinase; MLKL, mixed lineage kinase domain-like.

Research has unveiled significant crosstalk among initiators, executors and implementers of ferroptosis, pyroptosis and necroptosis (52). These three forms of non-apoptotic RCDs are extensively studied, each characterised by distinct molecular traits (53). This section offers a concise overview of the mechanisms and roles of these key forms of non-apoptotic RCDs (**Figure 2**).

### 3.1 Ferroptosis

Ferroptosis is a unique iron-dependent and oxidative form of cell death (6). Compared to other types of cell death, ferroptosis exhibits marked variations in genetic, biochemical, morphological and metabolic traits (1, 54). Notably, ferroptosis can rapidly propagate within cell populations in a wave-like fashion. Morphologically, cells undergoing ferroptosis display mitochondrial abnormalities including swelling, altered density and rupture of the outer membrane. Uncontrolled lipid peroxidation, driven by iron and Fenton-like reactions, disrupts lipid membranes, a hallmark of ferroptosis. Ultimately, the pathways related to iron, glutathione (GSH) and lipid metabolism intersect to regulate ferroptosis execution (55).

Several cellular processes regulate ferroptosis (**Figure 2A**). Ferroptosis is triggered by the excessive oxidative damage (peroxidation) of lipids in the cell membranes, dependent on iron, reactive oxygen species (ROS) and phospholipids containing polyunsaturated fatty acids (PUFAs) (56–58). Intracellular PUFAs are enzymatically converted into PUFA-phospholipid-peroxides (PUFA-PL-OOH), leading to lipid peroxide buildup within cellular membranes (59, 60). Iron-mediated lipid peroxidation is regulated by four antioxidant pathways linked to ferroptosis: System Xc–GPX4, FSP1-CoQ10, DHODH/CoQH2 and GCH1-BH4. The antiporter System Xc-, encompassing SLC7A11 and SLC3A2, facilitates cystine absorption, essential for GSH production (61–65). Notably, erastin, a common ferroptosis inducer, can suppress the expression of SLC7A11, leading to the dysfunction of System Xc-. This leads to inhibited cystine uptake, reduced GSH production and ultimately ferroptosis activation (66). Excessive iron, stored as ferritin is released upon cellular stimulation and undergoes degradation, resulting in Fe^3+^ release in large quantities. This Fe^3+^ is then converted to Fe^2+^ by the transferrin receptor-1 (TFR1) and ultimately discharged into the cytoplasmic iron pool. Consequently, ROS levels increase, leading to detrimental effects (67–69). GPX4 assumes a crucial role in safeguarding against ferroptosis by reducing ROS cellular levels and proficiently mending lipid oxidation-induced cellular damage. Nevertheless, GPX4 inactivation due to GSH depletion and direct inhibition via RSL3 or FIN56 comprises the antioxidant capacity and subsequent lipid ROS overproduction, consequently triggering ferroptosis through uncontrolled lipid peroxidation (70–72). Ferroptosis can be initiated by disrupting the System Xc- cystine/glutamate antiporter or GPX4, resulting in impaired redox balance within the GSH system. Additionally, ferroptosis suppressor protein 1 (FSP1) serves as another inhibitor of ferroptosis (73, 74). The FSP1-CoQ10-NAD(P)H pathway operates independently of GPX4 and GSH to suppress phospholipid peroxidation and ferroptosis (61). In the DHODH/CoQH2 pathway, DHODH functions concomitantly with mitochondrial GPX4 (yet not aligned with cytosolic GPX4 or FSP1) in hindering ferroptosis within the inner mitochondrial membrane. This inhibition is achieved through the reduction of ubiquinone to ubiquinol, which serves as a radical-trapping antioxidant possessing anti-ferroptosis properties (63). In another ferroptosis pathway, GTP cyclohydrolase-1 (GCH1) serves as the primary enzyme regulating the production of BH4. Furthermore, by genetically or pharmacologically inhibiting GCH1, levels of BH4 can be depleted, thereby facilitating erastin-induced cell death, enhancing lipid peroxidation and promoting ferrous iron accumulation, ultimately resulting in ferroptosis (75). A recent research found that 7-DHC attenuates ferroptosis by diverting the peroxidation pathway from phospholipids and protects cells from phospholipid peroxidation at the cell membrane and mitochondria. However, high levels of 7-DHC also lead to more aggressive cancer manifestations and promote cancer metastasis (76, 77). Notably, ferroptosis governs the growth and proliferation of various tumour cell types, including lymphocytoma, pancreatic ductal cell carcinoma, renal cell carcinoma and hepatocellular carcinoma (HCC) (78–81).

### 3.2 Pyroptosis

Pyroptosis, a programmed inflammatory cell death mechanism, involves the cleavage and activation of gasdermin, resulting in compromised cell membrane integrity and the activation of inflammasome sensors, releasing cellular proteins as danger signals (82, 83). Despite certain similarities with apoptosis, such as DNA damage and chromatin state changes, pyroptosis exhibits distinct morphological traits (82, 84). Cells undergoing pyroptosis exhibit swelling and bubble-shaped protrusions on the cellular membrane before eventual rupture, contrasting with the regulated and non-inflammatory nature of apoptosis (85). Notably, pyroptosis elicits inflammation in response to various extrinsic and intrinsic stimuli, including microbial pathogens, toxins and chemotherapeutic agents (86). Compared to the abrupt rupture seen in necrosis, pyroptosis results in cytoplasmic flattening due to the leakage of the plasma membrane (7).

Previously, researchers speculated that pyroptosis arises as a response to bacterial infection, which was primarily mediated by caspase-1 in monocytes. However, recently, caspase-11/4/5 has been demonstrated to play a role in sensing intracellular lipopolysaccharide (LPS) and expanding the range of pyroptosis mediators. This finding indicates that pyroptosis is not specific to a particular cell type (87). Recent research has revealed that gasdermin D (GSDMD), a substrate for caspase-1 and caspase-11/4/5, acts as the executioner of pyroptosis by forming membrane pores (83, 85). During pyroptosis, the pore-forming domains of gasdermin-N are separated from the repressor domains of gasdermin-C. The gasdermin-N domains subsequently oligomerise and create pores in the cell membrane, resulting in compromised membrane integrity and the induction of cell pyroptosis (7, 88).

Pyroptotic cells are orchestrated through two primary molecular pathways: the classical Caspase-1-dependent route and the non-Caspase-1-dependent pathway. Caspase 1 governs the canonical pyroptosis pathway, while caspases 4, 5 and 11 mediate the non-canonical pyroptosis pathway (**Figure 2B**). In the canonical pathway, various stimuli such as viruses, bacteria, toxins, ATP or ROS, which are known as pathogen-associated molecular patterns (PAMPs) or damage-associated molecular patterns (DAMPs), trigger the activation of specific inflammasome sensors, including NOD-like receptor family pyrin domain-containing 1B (NLRP1b), NOD-like receptor family pyrin domain-containing 3 (NLRP3), NOD-like receptor family CARD domain-containing protein 4 (NLRC4), AIM2 and pyrin (89–91). Upon activation, the sensors of the inflammasome engage in direct or indirect interactions that activate caspase-1. Subsequently, caspase-1 cleaves the N-terminal end of GSDMD, thereby forming pores on the cell membrane (87). This process ultimately culminates in the release of cytoplasmic contents and the onset of cellular pyroptosis. Furthermore, caspase-1 catalyses the dissociation of pro-IL-1β and pro-IL-18, resulting in mature IL-1β and IL-18 (92). In the non-canonical pathway, the LPS derived from Gram-negative bacteria interacts with either CASP11 or CASP4/5, leading to the activation of the inflammasome (93). Subsequently, the inflammasome activation induces the cleavage and generation of the N-terminal fragment of GSDMD (GSDMD-N), which facilitates pyroptotic cell death by forming pores on the plasma membrane through its pore-forming activity (94–97). Pyroptosis is considered pro-inflammatory due to its ability to release DAMPs from expired cells and promote the maturation and secretion of interleukin-1 family members, such as IL-1β and IL-18, through inflammasome activation (98). A novel discovery has shown that GSDMD activation during pyroptosis could be spatiotemporally modulated by a palmitoylation-depalmitoylation relay model (99). Some studies have demonstrated that caspase-3 can modulate and activate GSDME, leading to pyroptosis (86). Recent research has found that USP48 can facilitate pyroptosis by stabilising GSDME and sensitising cancer cells to pyroptosis (100). Current studies have uncovered that pyroptosis is linked with the proliferation and migration of multiple cancers, including colon cancer, osteosarcoma, head and neck squamous cell carcinoma and breast cancer (101–104).

### 3.3 Necroptosis

Necroptosis is another type of immunogenic cell death (ICD), which is a caspase-independent RCD process triggered by infection. It is characterised by the phosphorylation of pseudokinase mixed lineage kinase domain-like (MLKL) by receptor-interacting serine-threonine kinase 3 (RIPK3) (105–107). Morphologically, necroptosis involves organelle swelling, plasma membrane rupture, cytoplasm and nucleus breakdown, and the release of intracellular components known as DAMPs, which propagate secondary inflammation. This pro-inflammatory nature suggests that necroptosis may have evolved as an innate immune process that supplements apoptosis in pathogen clearance (74, 108, 109). Under conditions of insufficient apoptosis, necroptosis is regulated by distinct death receptors (DRs) like FAS and tumour necrosis factor receptor 1 (TNFR1) or pattern recognition receptors (PRRs) such as toll-like receptor 3 (TLR3), which sense detrimental cues from the intracellular and extracellular environments to trigger necroptosis (110–112) (**Figure 2C**).

In the necroptosis process, death-inducing molecules such as FasL and TRAIL stimulate necrosome complex assembly. NF-κB inhibition induces Complex II a (RIPK1-independent) formation, which is composed of TRADD, FADD and caspase-8 (113). Moreover, the induction of complex IIb, which comprises RIPK1, FADD and caspase-8, is observed when RIPK1 ubiquitination is inhibited or when cytotoxicity induces phosphorylation, which indicates that the initial stages of TNF signalling are hindered. Inhibition of caspase-8 leads to necrosome complex formation through RIPK1 and RIPK3 homotypic interactions, involving the RIP homotypic interaction motif and consequently activating MLKL through phosphorylation (109). Intracellular adaptor molecules, such as FADD, recruit RIPK1 and subsequently RIPK3, forming the necrosome through phosphorylation processes. The necrosome is a protein complex comprising core components such as RIPK1, RIPK3 and MLKL (105, 112, 114). The translocation of MLKL to the membrane is imperative for facilitating the influx of Ca^2+^, an early occurrence in TNF-induced necroptosis (115). During TNFR1 stimulation, a critical component of necroptosis, known as the necrosome, assembles. In this process, CASP8, which is typically involved in apoptosis, is suppressed. Upon TNFα stimulation, the intracellular tails of TNFR1 recruit various proteins that collectively form a signalling complex called ‘Complex I’. In general, the activation of RIPK1 kinase by TNFα, FASL (FAS ligand) and TRAIL (tumour necrosis factor-associated apoptosis-inducing ligand) initiates the formation of a complex known as inhibitors of apoptosis (cIAPs) at the cell membrane. This cascade reaction ultimately triggers the activation of NF-κB, thereby promoting cell survival (8, 116, 117). Necroptosis plays indispensable roles in various physiological functions, but dysregulation is associated with multiple human diseases (118). In addition to their essential roles in physiological processes, molecules associated with necroptosis contribute to the development of various cancers including breast cancer, liver cancer, PAAD and CRC (106, 118–121). Recent findings highlight the crucial involvement of necroptosis in tumour development and metastasis, suggesting promising prospects for leveraging necroptosis in cancer treatment (8).

## 4 LncRNAs regulate ferroptosis, pyroptosis and necroptosis

**Figure 3:**
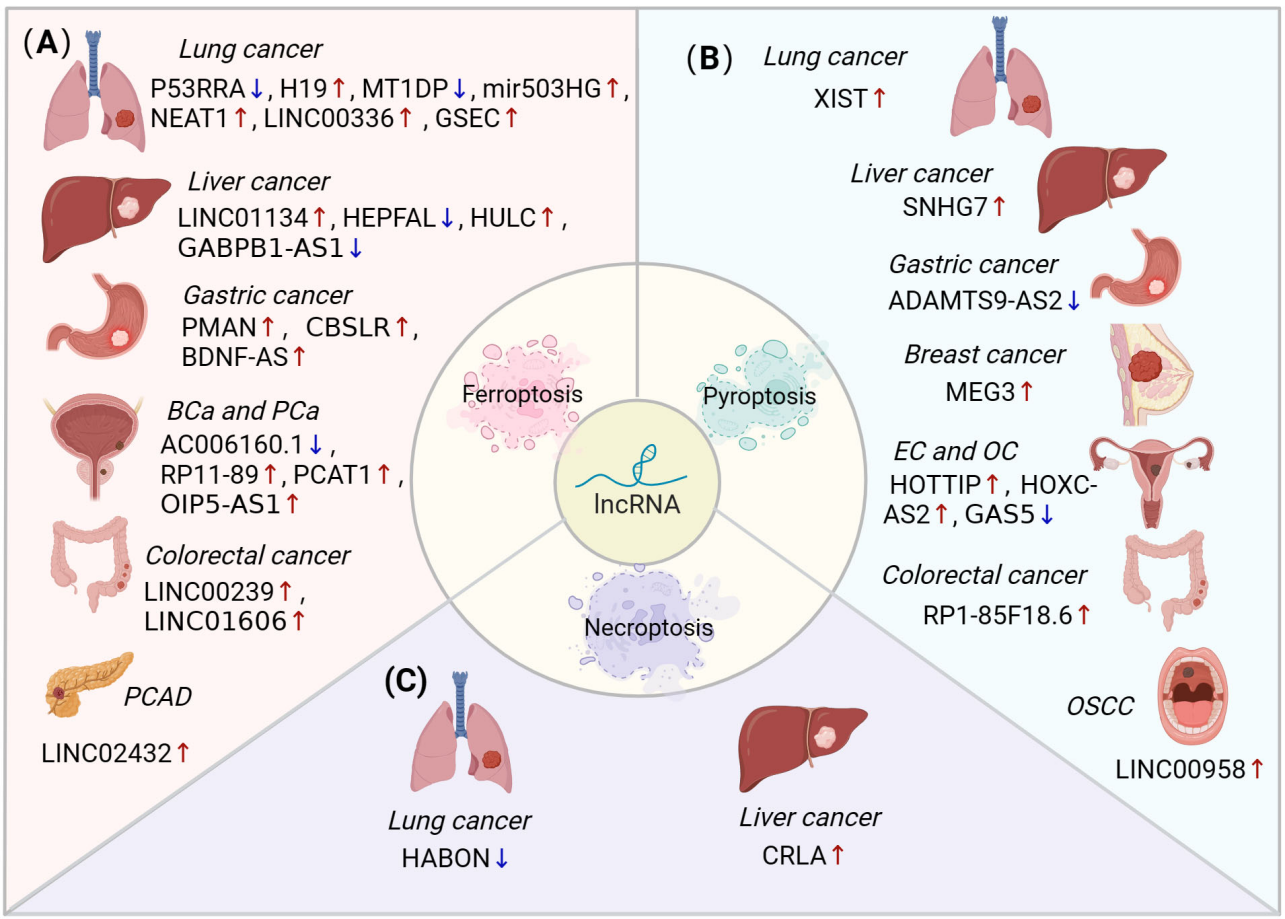
The role of lncRNA-mediated ferroptosis, pyroptosis and necroptosis in different cancer types. **(A)** LncRNAs can regulate ferroptosis to influence various types of cancers, including lung, liver, stomach, bladder, prostate, colorectal, and pancreatic cancers. **(B)** LncRNAs mediate pyroptosis in lung, liver, gastric, breast, endometrial, ovarian, colorectal, and oral squamous cell carcinomas. **(C)** The dysregulation of lncRNAs inhibits necroptosis in lung and liver cancers. BLCA, bladder cancer; PCa, prostatic cancer; PAAD, pancreatic adenocarcinoma; EC, endometrial cancer; OC, ovarian cancer; OSCC, oral squamous cell carcinoma.

Emerging studies uncovered that lncRNAs influenced tumour progression by regulating non-apoptotic RCDs. In the following sections, the role of lncRNA-regulated ferroptosis, pyroptosis and necroptosis in different cancer types are discussed, including lung cancer, liver cancer, gastric cancer (GC), breast cancer (BC), bladder cancer (BLCA), colorectal cancer (CRC), endometrial cancer (EC), ovarian cancer (OC), pancreatic adenocarcinoma (PAAD), prostatic cancer (PCa), oral squamous cell carcinoma (OSCC) and glioma (**Figure 3**, **Table 1**).

**Table 1:** Examples of non-apoptotic regulated cell death (RCD) regulated by long non-coding RNAs (lncRNAs) in cancers.

lncRNA	Interacting molecules	Axis/Signaling Pathways	Anti-/Pro-non-apoptotic RCDs types	Function	Cancer	Reference
LINC00336	miR-6852	CBS	Anti-ferroptosis	Inhibits ferroptosis in lung cancer by sponging miR-6852	Lung cancer	(122)
H19	miR-19b-3p	FTH1	Anti-ferroptosis	H19 inhibits ferroptosis via the miR-19b-3p/FTH1 axis under curcumenol treatment.	Lung cancer	(123)
mir503HG	miR-1273c	SOX4	Anti-ferroptosis	Inhibits NSCLC via sponging miR-1273c and regulating SOX4 expression.	NCSLC	(124)
P53RRA	G3BP1	— —	Pro-ferroptosis	Promotes ferroptosis and apoptosis in cancer via nuclear sequestration of p53.	Lung and breast cancers	(125)
MT1DP	miR-365a-3p	NRF2	Pro-ferroptosis	Ectopic expression of MT1DP sensitized A549 and H1299 cells to erastin-induced ferroptosis through downregulation of NRF2;	NCSLC	(125)
GSEC	miR-101-3p	CISD1	Pro-ferroptosis	Inhibit the proliferative and migratory capabilities of LUAD cells by miR-101-3p/CISD1 axis	LUAD	(126)
NEAT1	ASCL4	— —	Pro-ferroptosis	Regulates ferroptosis sensitivity in NSCLC	NCSLC	(127)
LINC01134	Nrf2	GPX4	Anti-ferroptosis	Inhibits ferroptosis through GPX4 in Hepatocarcinoma and inhibits sensitivity of oxaliplatin	HCC	(128)
HEPFAL	— —	— —	Pro-ferroptosis	Promotes ferroptosis by mediating SLC7A11 ubiquitination	HCC	(129)
GABPB1-AS1	GABPB1	— —	Pro-ferroptosis	Causes the accumulation of ROS during erastin-induced ferroptosis in HepG2 cells	HCC	(130)
HULC	miR-3200-5p	ATF4	Anti-ferroptosis	Downregulation of HULC induces liver cancer cell ferroptosis by targeting the miR-3200-5p/ATF4 axis to modulate the development of HCC	HCC	(131)
PMAN	HIF-1α	— —	Anti-ferroptosis	Inhibits ferroptosis by promoting the cytoplasmic translocation of ELAVL1 in peritoneal dissemination from GC	GC	(19)
CBSLR	m6A-YTHDF2	— —	Anti-ferroptosis	Suppresses ferroptosis through m6A-YTHDF2-dependent modulation of CBS in GC.	GC	(21)
BDNF-AS	WDR5	FBXW7	Anti-ferroptosis	regulates ferroptosis in GC by affecting VDAC3 ubiquitination	GC	(132)
LINC00239	Keap1	— —	Anti-ferroptosis	inhibits ferroptosis in CRC by binding to Keap1 to stabilize Nrf2	CRC	(133)
LINC01606	miR-423-5p	SCD1-Wnt/β-catenin-TFE3	Anti-ferroptosis	Protects colon cancer cells from ferroptotic cell death and promotes stemness by SCD1-Wnt/β-catenin-TFE3 feedback loop signalling	CRC	(134)
LINC02432	Hsa-miR-98-5p	HK2	Anti-ferroptosis	Inhibits ferroptosis and predicts immune infiltration, tumour mutation burden, and drug sensitivity in PAAD.	PAAD	(135)
OIP5-AS1	miR-128-3p	SLC7A11	Anti-ferroptosis	Inhibits ferroptosis under chronic Cd exposure by targeting miR-128-3p/SLC7A11 signaling	PCa	(136)
PCAT1	TFAP2C	c-Myc/miR-25-3p/SLC7A11	Anti-ferroptosis	Inhibits ferroptosis in docetaxel-resistant prostate cancer	PCa	(137)
RP11-89	miR-129-5p/PROM2	— —	Anti-ferroptosis	induces tumour cell proliferation and migration, promotes tumorigenesis and inhibits cell cycle arrest and inhibits ferroptosis via PROM2-activated iron export	BLCA	(138)
AC006160.1	— —	— —	Pro-ferroptosis	protective factor for the progression of BLCA.	BLCA	(139)
TUG1	MAZ	TUG1/MAZ/FTH1 Axis	Anti-ferroptosis	Attenuates the antiglioma effect of dihydroartemisinin by inhibiting ferroptosis.	Glioma	(140)
NEAT1	SLC7A11	GPX4	Anti-ferroptosis	NEAT1 overexpression suppresses GNA inhibition on cell vitality and eliminates GNA-induced melanoma cell ferroptosis.	Melanoma	(127)
MEG8	miR-497-5p	miR-497-5p/NOTCH2 axis	Pro-ferroptosis	Inhibits the proliferation and induces the ferroptosis of hemangioma endothelial cells by regulating miR-497-5p/NOTCH2 axis.	Hemangioma	(141)
HOXC-AS2	miR-876-5p	HKDC1	Anti-pyroptosis	Increases pyroptosis of EC cells under HG conditions via HOXC-AS2/miR-876-5p/HKDC1 axis.	EC	(142)
SNHG7	miR-34a	miR-34a/SIRT1	Anti-pyroptosis	Inhibits NLRP3-dependent pyroptosis via miR-34a/SIRT1 axis in liver cancer.	HCC	(143)
HOTTIP	miR-148a-3p	miR-148a-3p/Akt2	Anti-pyroptosis	Promotes OC proliferation and suppresses NLRP1 inflammasome-dependent pyroptosis through miR-148a-3p/Akt2 signaling pathway.	OC	(144)
RP1-85F18.6	— —	ΔNp63 and GSDMD	Anti-pyroptosis	Downregulation of RP1-85F18.6 promotes LDH release, induces GSDMD cleavage and cell membrane rupture, leading to CRC cell pyroptosis	CRC	(145)
lncOR7C2-1	EPS15L1	GSDME	Anti-pyroptosis	The chimeric protein EPS15L1-lncOR7C2-1 can promote tumours by regulating GSDME-dependent pyroptosis	Breast cancer	(146)
GAS5	— —	— —	Pro-pyroptosis	Inhibited OC cell proliferation by activating cell pyroptosis	OC	(147)
LINC00958	miR-4306	miR-4306/AIM2	Pro-pyroptosis	Induces AIM2-dependent pyroptosis via miR-4306/AIM2 axis and enhanced OSCC cell proliferation by downregulating SIRT1 to decrease p53 expression.	OSCC	(148)
ADAMTS9-AS2	miR-223-3p	miR-223-3p/NLRP3	Pro-pyroptosis	Acted as a tumour suppressor and enhanced cisplatin sensitivity in GC cells by activating NLRP3 mediated pyroptosis through sponging miR-223-3p	GC	(149)
MEG3	— —	NLRP3/caspase-1/GSDMD	Pro-pyroptosis	Induces pyroptosis via the NLRP3/caspase-1/GSDMD pathway under cisplatin treatment	BRCA	(150)
XIST	miR-335	miR-335/SOD2/ROS	Pro-pyroptosis	Knockdown of LncRNA-XIST inhibited NSCLC progression by triggering miR-335/SOD2/ROS signal pathway mediated pyroptotic cell death	NSCLC	(151)
HABON	mPTP	— —	Anti-necroptosis	Upregulated by TCF-4 and inhibited RIPK1-induced necroptosis in the mesenchymal-like LUAD cells by impairing of RIPK1–RIPK3 interaction via binding to the ID of RIPK1.	Liver cancer	(152)
lncCRLA	— —	— —	Anti-necroptosis	Inhibits RIPK1-induced necroptosis by impairing RIPK1RIPK3 interaction via binding to the intermediate domain of RIPK1	LUAD	(153)
LncRNA-107053293	miR-148a-3p	miR-148a-3p/FAF1 axis	Pro-necroptosis	Regulated necroptosis by acting as a ceRNA of miR-148a-3p. FAF1, as a gene target of miR-148a-3p, also affects necroptosis.	Chicken trachea cell	(154)

### 4.1 LncRNAs regulate ferroptosis

Ferroptosis is intricately linked to cancer progression. However, the underlying mechanisms, particularly those involving lncRNA-mediated ferroptosis in oncogenesis, remain insufficiently explored. This section focuses on the lncRNAs that regulate ferroptosis across various cancers through via diverse mechanisms (**Figure 3A**).

#### 4.1.1 LncRNAs regulate ferroptosis in lung cancer

Lung cancer remains the leading cause of cancer-related mortality globally, with non-small cell lung cancer (NSCLC) representing over 80% of all cases (155). Recent studies have unveiled that lncRNAs can either promote or inhibit ferroptosis, thereby influencing lung cancer progression. Ferroptosis plays a significant role in tumorigenesis depression by selectively eliminating cells experiencing a nutrient deficiency or damage caused by environmental or infection-induced stress (156). For instance, LINC00336 acts as a competing endogenous RNA (ceRNA) to suppress ferroptosis in lung cancer. The inactivation of p53 by lymphoid-specific helicase leads to the induction of ELAV-like RNA binding protein 1 expression, which, in turn, enhances the levels of LINC00336 through transcriptional regulation. Subsequently, LINC00336 acts as a ceRNA by sequestering miR-6852, ultimately resulting in increased CBS mRNA levels. This molecular cascade promotes cell proliferation and tumour growth, while concurrently inhibiting ferroptosis in lung cancer (122). As another example, lncRNA H19 counteracts the anticancer properties of curcumenol via the miR-19b-3p/FTH1 axis in lung cancer. H19 suppresses lipid ROS generation while promoting GSH production, thereby impeding ferroptosis (123). The reduction in lncRNA MIR503HG levels, influenced by XAV939, may suppress NSCLC by serving as a sponge for miR-1273c and controlling SOX4 expression. Additionally, the decrease in SLC7A11 expression, which is triggered by XAV939, may hinder NSCLC progression through the ferroptosis pathway (124).

Moreover, lncRNA can also facilitate ferroptosis in lung cancer. For example, the tumour-suppressive lncRNA P53RRA, also referred to as LINC00472, is downregulated in various cancers, such as lung, liver, breast and colon cancers. In lung cancer, P53RRA interacts with Ras GTPase-activating protein-binding protein 1 (G3BP1) within the cytosol, resulting in the displacement of p53 from the G3BP1 complex. As a result, p53 is localised to the nucleus, resulting in cell cycle arrest, apoptosis activation and ferroptosis promotion. P53RRA facilitates ferroptosis by influencing the transcription of various metabolic genes, including the inhibition of SCL7A11. Furthermore, P53RRA enhances erastin-induced ferroptosis and increases lipid ROS and iron levels in cells (125). A recent study reported a new approach wherein targeting the MT1DP/miR-365a-3p/NRF2 pathway facilitated erastin-induced ferroptosis. The use of erastin/MT1DP@FA-LPs (E/M@FA-LPs) effectively intensified ferroptosis, leading to reduced cellular GSH levels and increased lipid ROS. Moreover, the ectopic expression of the metallothionein 1D pseudogene (MT1DP) enhanced the susceptibility of A549 and H1299 cells to erastin-induced ferroptosis by suppressing NRF2. Additionally, MT1DP overexpression resulted in elevated levels of malondialdehyde (MDA) and ROS. Notably, in instances of cancer cells being exposed to erastin, an evident increase in intracellular ferrous iron levels has been noted along with a decline in GSH concentrations. Conversely, downregulating MT1DP demonstrated the opposite effect (126). In lung adenocarcinoma (LUAD), ferroptosis-related lncRNA GSEC expression was increased, while miR-101-3p expression was decreased. Moreover, GSEC can influence ferroptosis by sequestering miR-101-3p, thereby influencing LUAD progression (157). Another study investigated the involvement of nuclear-enriched transcript 1 (NEAT1) has been reported to influence ferroptosis, exhibiting susceptibility to erastin-induced ferroptosis (127).

#### 4.1.2 LncRNAs regulate ferroptosis in liver cancer

Liver cancer is the leading cause of death from malignancies globally, underscoring the urgent need for new treatment options for patients (158, 159). Numerous studies have shown that lncRNAs can either promote or inhibit ferroptosis in liver cancer through various mechanisms, including transcriptional regulation, protein modification and sequestering miRNA. For instance, Kang et al. revealed that LINC01134 suppresses ferroptosis by facilitating Nrf2 protein recruitment to the promoter region of the GPX4 gene, thereby promoting GPX4 transcription and enhancing liver cancer resistance to OXA (128). Another study indicated that lncRNA HEPFAL contributes to ferroptosis in hepatoma cells by facilitating the ubiquitination of SLC7A11. Thereby, HEPFAL emerges as a promising candidate for the diagnosis and therapeutic intervention of HCC (129). A recent study has revealed that URB1-AS1 attenuates sorafenib-induced ferroptosis by facilitating ferritin phase separation and decreasing the intracellular free iron level. Moreover, it was discovered that specifically silencing the expression of URB1-AS1 with N-acetylgalactosamine (GalNAc)-small interfering URB1-AS1 effectively potentiated the sensitivity of HCC cells to sorafenib in an *in vivo* tumour model. This suggests that URB1-AS1 targeting may represent a potential therapeutic approach to overcome sorafenib resistance in HCC (160).

Erastin-induced upregulation of lncRNA GABPB1-AS1 promotes the formation of RNA duplexes with GABPB1 mRNA, leading to the inhibition of GABPB1 translation. This inhibition ultimately results in reduced expression of PRDX5, leading to ROS accumulation. Therefore, GABPB1-AS1 lncRNA could potentially contribute significantly to erastin-induced ferroptosis in HCC (130). Another study found that liver cancer cell ferroptosis can be induced by suppressing HULC, mediated by the miR-3200-5p/ATF4 axis. This regulatory pathway emerges as a crucial determinant in the pathogenesis of HCC (131).

#### 4.1.3 LncRNAs regulate ferroptosis in gastrointestinal tumours

GC, the fifth most prevalent cancer worldwide, is the third highest cause of cancer-related deaths (161, 162). LncRNAs can influence ferroptosis by interacting with different regulators, thereby catalysing the initiation and progression of gastrointestinal tumours. For example, in GC, hypoxia-induced HIF-1α and lncRNA-PMAN facilitate the cytoplasmic translocation of ELAVL1 during peritoneal dissemination. This orchestration effectively inhibits ferroptosis, as PMAN, upregulated by HIF-1α, stabilises SLC7A11 mRNA through ELAVL1-mediated cytoplasmic distribution. The resulting accumulation of SLC7A11 elevates l-GSH levels, inhibiting ROS and iron accumulation, thereby shielding GC cells from ferroptosis induced by agents like erastin and RSL3 (19). Additionally, hypoxia-induced signalling pathways, including CBSLR, CBS and ACSL4, modulate the metabolism of PUFAs, conferring resistance to ferroptosis in GC cells. Under hypoxic conditions, CBSLR is transactivated by HIF-1α, leading to the modulation of ferroptosis in GC cells. Moreover, the increased expression of CBSLR in GC tissues correlates with an unfavourable prognosis and reduced responsiveness to chemotherapy, underscoring its potential as a prognostic biomarker and a determinant of chemotherapy efficacy (21). As another example, the BDNF-AS/WDR5/FBXW7 axis regulates ferroptosis in GC by influencing the ubiquitination of VDAC3. Furthermore, BDNF-AS demonstrates promising prospects as both a prognostic biomarker and a therapeutic target in GC (132).

In CRC, which ranks as the second leading cause of cancer-related deaths globally, lncRNAs like LINC00239 fosters CRC cell proliferation by interacting with Keap1, thereby disrupting the Keap1/Nrf2 complex and consequently bolstering Nrf2 protein stability, which results in its translocation to the nucleus (163, 164). The overexpression of LINC00239 suppresses ferroptosis by interacting with the Kelch domain (Nrf2-binding site) of Keap1, thereby blocking Nrf2 ubiquitination and augmenting its protein stability. LINC00239 promotes CRC tumorigenesis *in vitro* and *in vivo* (133). Similarly, LINC01606 inhibits ferroptosis and promotes CRC stemness via the SCD1-Wnt/β-catenin-IGHM enhancer 3 (TFE3) positive feedback loop signalling. Thus, LINC01606 emerges as a pivotal therapeutic target for CRC treatment (134).

Furthermore, LINC02432 exert an oncogenic role in PAAD by inhibiting ferroptosis through the miR-98-5p/HK2 axis. Moreover, its potential as a predictive marker for immune infiltration, drug responsiveness and tumour mutation burden underscores its significance. Further research is warranted to elucidate the precise mechanism underlying LINC02432 impact on PAAD prognosis (135).

#### 4.1.4 LncRNAs regulate ferroptosis in other cancers

In other cancers, lncRNAs also play crucial roles in initiating and progressing tumours by affecting ferroptosis. For instance, in PCa cells exposed to chronic Cd, the expression of lncRNA OIP5-AS1 is significantly elevated. OIP5-AS1 enhances cell proliferation and inhibits ferroptosis upon chronic Cd exposure by modulating the miR-128-3p/SLC7A11 pathway. Furthermore, targeting OIP5-AS1 holds promise as a viable therapeutic approach for managing Cd-induced progression of PCa (136). Another example is TFAP2C-regulated lncRNA PCAT1, which suppresses ferroptosis in docetaxel-resistant PCa via c-Myc/miR-25-3p/SLC7A11 pathway (137).

In BLCA, the lncRNA RP11-89 exerts an inhibitory effect on ferroptosis by facilitating PROM2-mediated iron export through its interaction with miR-129-5p. Moreover, the miR-129-5p/PROM2 axis is utilised by RP11-89 to promote tumour cell proliferation and migration, boost tumorigenesis and hinder cell cycle arrest. RP11-89 upregulates PROM2 expression and functions as a ceRNA targeting miR-129-5p (138). Additionally, the lncRNA AC006160.1 is a ferroptosis-related lncRNA that demonstrates significant potential in accurately predicting survival outcomes, clinical stages, tumour grades, immune cell infiltration and immune checkpoint expression in BLCA. Moreover, AC006160.1 exhibits a protective role in hindering BLCA progression (139).

In glioma, the lncRNA TUG1 undermines the antiglioma efficacy of dihydroartemisinin (DHA) by suppressing ferroptosis through the MAZ/FTH1 axis. Modulating TUG1 expression or inhibiting FTH1 can augment the antiglioma effects of DHA, presenting a promising strategy to enhance DHA’s effectiveness against glioma (140). As another example, SNAI3-AS1 interacted with SND1 in a competitive manner, disrupting the m6A-dependent recognition of Nrf2 mRNA 3’UTR by SND1, thereby reducing the mRNA stability of Nrf2. This, in turn, enhanced the anti-tumour efficacy of erastin *in vitro* and *in vivo* by facilitating ferroptosis, providing a theoretical basis for inducing ferroptosis to improve the treatment of glioma (165). Furthermore, the downregulation of NEAT1 inhibits the direct interaction between SLC7A11, indirectly inhibiting GPX-4 activity and facilitating ferroptosis. Moreover, NEAT1 overexpression counteracts GNA-mediated suppression of cell viability and prevents GNA-induced ferroptosis in melanoma cells (166). Another study investigated the impact of lncRNA MEG8 downregulation on haemangioma endothelial cells and found that decreased expression of lncRNA MEG8 inhibited cell proliferation and stimulated the occurrence of ferroptosis. Additionally, they observed that these effects were mediated through the modulation of the miR-497-5p/NOTCH2 signal pathway (141).

In conclusion, lncRNAs can affect the development of cancers by regulating the ferroptosis inducer ROS, the key regulatory factor Nrf2, SLC7A11, and the NF-κB signalling pathway. Therefore, an in-depth exploration of the molecular mechanism of lncRNA-regulated ferroptosis in tumours will illuminate new insight on the prevention and treatment of cancers.

### 4.2 LncRNAs regulate pyroptosis

Pyroptosis can suppress tumour initiation and progression, it also has the potential to create a microenvironment that favours tumour growth. LncRNAs can influence the initiation and progression of various cancers by interacting with various regulators to modulate pyroptosis (**Figure 3B**). This section describes the signalling pathways associated with lncRNA-mediated pyroptosis, which may represent a potential approach for regulating pyroptosis in cancers.

#### 4.2.1 LncRNAs regulate pyroptosis in gastrointestinal tumours

In CRC, silencing of lncRNA RP1-85F18.6 facilitates the induction of pyroptosis in CRC cells by escalating the discharge of LDH, promoting the fragmentation of GSDMD and disrupting the cell membrane, thereby impeding CRC cell proliferation and invasion. Additionally, lncRNA RP1-85F18.6 can inhibit GSDMD activity by enhancing ΔNp63 expression, thereby inhibiting the pyroptosis of CRC cells and facilitating the progression of cancer (145). The upregulation of ADAMTS9-AS2 suppresses GC advancement and enhances the sensitivity of cisplatin-resistant GC cells to cisplatin by mediating the miR-223-3p/NLRP3 axis, thereby inducing pyroptosis (149).

#### 4.2.2 LncRNAs regulate pyroptosis in OC

It has been demonstrated that HOTTIP is highly expressed in OC tissue samples and cell lines. The inhibition of HOTTIP inhibits the progression of OC and the initiation of pyroptosis regulated by the NLRP1 inflammasome through the miR-148a-3p/Akt2 signalling pathway (144). Likewise, lncRNA growth arrest-specific transcript 5 (GAS5) induces the formation of the inflammasome and promotes the inflammatory process by interfering with the glucocorticoid receptor. LncRNA GAS5 acts as a tumour suppressor and could be used as a potential therapeutic target for the diagnosis and treatment of OC (147).

#### 4.2.3 LncRNAs regulate pyroptosis in other cancers

In triple-negative breast cancer (TNBC), the inhibition of MEG3 not only partially mitigated the stimulatory effect of DDP on the NLRP3/caspase-1/GSDMD-mediated pyroptosis pathways, but also counteracted DDP’s inhibitory effect on tumour growth and metastasis, highlighting new avenues for the development of innovative therapeutic approaches for TNBC (150). HOXC‐AS2, a ceRNA, promotes pyroptosis and glycolysis through the miR‐876‐5p/HKDC1 signalling pathway, thereby stimulating the generation of inflammatory factors and the release of lactic acid into the TME, ultimately enhancing the proliferation and migration of EC cells (142). In liver cancer, lncRNA small nucleolar RNA host gene 7 (SNHG7) suppresses NLRP3-triggered pyroptosis through the miR-34a/SIRT1 pathway (143). LINC00958 also induces AIM2-dependent pyroptosis via the miR-4306/AIM2 axis and enhances OSCC cell growth by reducing SIRT1 levels, resulting in decreased p53 expression (148). Silencing of lncRNA-XIST hinders the proliferation of NSCLC via triggering the miR-335/SOD2/ROS axis-regulated cell pyroptosis (151). Moreover, emerging research indicates a potential correlation between the fusion of mRNA-lncRNA, leading to the production of the chimeric protein EPS15L1-lncOR7C2-1, which facilitates tumour growth by potentially controlling GSDME-related pyroptosis. These findings underscore the significance of lncRNA fusions in the regulation of tumour immunity via pyroptosis (146). Recent research has substantially refined our knowledge of the interplay between lncRNAs and pyroptosis in various cancers, offering significant insights that could enhance clinical strategies for the diagnosis and treatment of cancers.

### 4.3 LncRNAs regulate necroptosis

Mounting evidence underscores the role of lncRNA in either promoting or inhibiting necroptosis, thereby influencing tumour progression. In this section, we listed some lncRNAs linked with necroptosis in cancers (**Figure 3C**). For instance, lncCRLA, exhibits significant upregulation mediated by TCF-4 and suppresses RIPK1-induced necroptosis in mesenchymal-like LUAD cells. By impairing the interaction between RIPK1 and RIPK3, lncCRLA effectively obstructs necroptosis (153). Furthermore, HABON, a hypoxia-activated lncRNA, exerts a suppressive effect on necroptosis in liver cancer by binding with the mitochondria-related protein VDAC1. This interaction modulates the opening of the mitochondrial permeability transition pore, thereby regulating necroptosis (152). In addition, lncRNA-107053293 inhibits necroptosis induced by ammonia in trachea cells and LMH cells via miR-148a-3p/FAF1 axis. FAF1, as a gene target of miR-148a-3p, also affects necroptosis via its interaction with the necrosis-related genes RIPK1 and RIPK3 (154). Together, lncRNAs can affect necroptosis to influence the development of cancers by interacting with the molecular effectors RIPK3 and MLKL. Although lncRNA-mediated necroptosis are still limited to molecular biology and disease model experiments, the regulatory role of lncRNA-mediated necroptosis in cancers, as well as its participation in the pathophysiological process of various cancers reflected by lncRNA, are bound to be applied and reflected in future clinical studies. The scene will be set for lncRNA-mediated necroptosis to emerge prominently through early screening of high-risk groups for tumours and diagnosis and treatment of patients.

Taken together, lncRNAs exhibit oncogenic or anti-carcinogenic effects by various mechanisms to regulate non-apoptotic RCD genes and relative pathways involved in GSH, NLRP3 and RIPK3/MLKL. The above mentioned that a large number of lncRNAs have been implicated in hallmarks of cancer by promoting or inhibiting non-apoptotic RCDs. Therefore, non-apoptotic RCD associated lncRNAs could be used as promising diagnostic biomarkers and therapeutic targets in cancer treatment.

## 5 Potential clinical applications of lncRNAs in non-apoptotic RCDs

**Figure 4:**
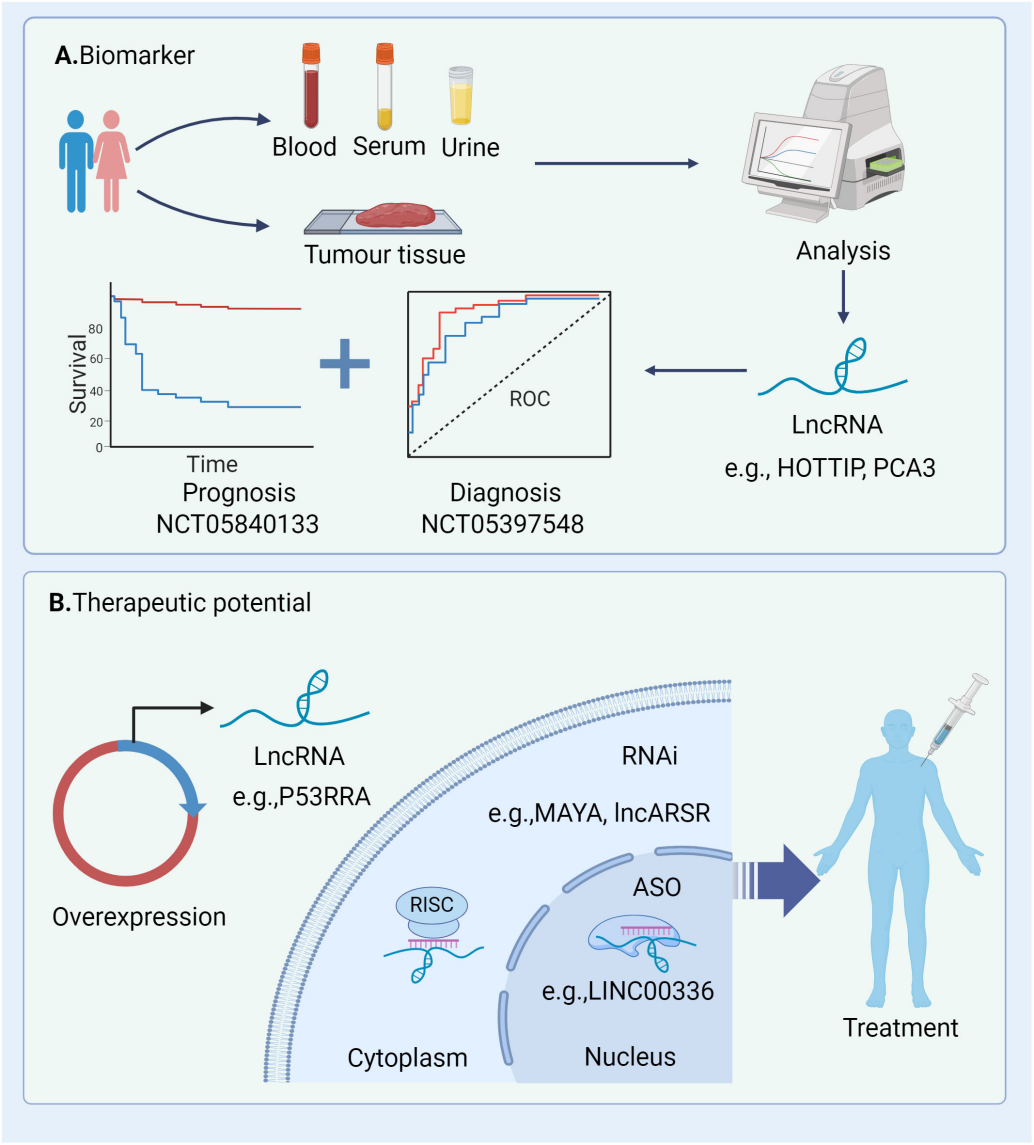
Potential clinical applications of lncRNAs in cancers. **(A)** These lncRNAs can be quantified and analysed from tumour tissue and liquid biopsy specimens (e.g. serum and urine) and thus have great potential as diagnostic and prognostic biomarkers; **(B)** Strategies for modulating lncRNAs within the cytoplasm and nucleus encompass knockdown and overexpression techniques. Knockdown methodologies comprise RNA interference (RNAi) and antisense oligonucleotides (ASOs). RNAi can be achieved through chemically synthesized siRNAs or pooled via lentivirally delivered short hairpin RNAs (shRNAs). RNAi, RNA interference; ASO, antisense oligonucleotides; RISC, RNA-induced silencing complex; ROC, receiver operating characteristic.

Recent studies have highlighted the close association between lncRNAs and non-apoptotic RCDs processes, including ferroptosis, pyroptosis and necroptosis. These lncRNAs play pivotal roles in modulating RCD and related cell death pathways, thereby influencing cancer progression and the efficacy of clinical therapies (134, 167). Given their prevalence, functional significance, and expression specificity, there is growing interest in evaluating lncRNAs as novel biomarkers and therapeutic targets in clinical settings. In the following sections, we will delve into the involvement of lncRNAs in pathways leading to RCD across various tumour types. Additionally, we will explore the therapeutic potential of targeting lncRNAs involved in RCD as a promising strategy for cancer treatment (**Figure 4** **).**

### 5.1 The diagnostic and prognostic value of lncRNAs

A considerable number of lncRNAs is detectable in both traditional tumour biopsies and liquid biopsies (e.g. blood, serum, and urine), presenting potential as diagnostic and prognostic biomarkers (**Figure 4A**). The current clinical applications of lncRNAs functioning as biomarkers in different tumours are outlined in **Table 2**. These lncRNA biomarkers offer distinct advantages over traditional diagnostic markers in clinical settings. For instance, lncRNA prostate cancer antigen 3 (PCA3) has been established as a non-invasive initial diagnostic indicator for prostate cancer, demonstrating reliable test characteristics and clinical utility (NCT01632930) (168). Additionally, several clinical trials are currently underway to explore the diagnostic and prognostic potential of lncRNAs in various cancer types (e.g., NCT04269746, NCT05397548, NCT04729855).

**Table 2:** Examples of clinical trials exploring the application of lncRNAs in cancers.

NCT Number	Study Title	LncRNA	Cancer type	Primary Purpose	Interventions	Study Status
NCT04269746	Assessment Of Long Noncoding RNA CCAT1 In Colorectal Cancer Patients	CCAT1	CRC	Diagnosis	DIAGNOSTIC_TEST: CCAT1	COMPLETED
NCT05397548	Use of Circulating Exosomal LncRNA-GC1 to Monitor Gastric Cancer	LncRNA-GC1	GC	Prognostic biomarkers	DIAGNOSTIC_TEST: Measurement of levels of circulating exosomal lncRNA-GC1	COMPLETED
NCT04729855	Association of Autophagy-related Genes, LncRNA and SNPs With Colorectal Cancer in Egyptian Population	HOTTIP	CRC	Diagnostic biomarkers	DIAGNOSTIC_TEST: serum HOTTIP, EIF4EBP1 and serum SNP HOTTIP rs1859168	RECRUITING
NCT05141383	Comparative Study of Diagnostic and Prognosis Biomarkers of Prostate Cancer in Liquid Biopsy	— —	Prostate Cancer	Diagnostic and prognosis biomarkers	OTHER: Urine sampling	RECRUITING
NCT05270174	A Prospective, Multicenter Cohort Study of Urinary Exosome lncRNAs for Preoperative Diagnosis of Lymphatic Metastasis in Patients With Bladder Cancer	ELNAT1	BLCA	Prognosis and independent predictor	OTHER: no intervention	NOT_YET_RECRUITING
NCT05840133	Study of Long Non-coding RNA SNHG15 as a Novel Biomarker in HBV Associated HCC	SNHG15	HCC	Diagnostic biomarker	DIAGNOSTIC_TEST: No intervention was required for patients or control group in this study	NOT_YET_RECRUITING
NCT05749497	Prediction of the Chronicization of Radiation-induced Acute Intestinal Injury Based on the Expression Level of lncRNA	UCID, NEAT1, ciRS-7	Radiation-induced Intestinal Injury	Prognosis	OTHER: NCRT+TME	NOT_YET_RECRUITING
NCT03830619	Serum Exosomal Long Noncoding RNAs as Potential Biomarkers for Lung Cancer Diagnosis	— —	Lung Cancer	Prognostic biomarkers	DIAGNOSTIC_TEST: collect samples	COMPLETED
NCT05708209	The Long Non Coding MALAT1 as a Potential Salivary Diagnostic Biomarker in Oral Squamous Cell Carcinoma Through Targeting mi RNA 124	MALAT1	OSCC	Diagnostic biomarker	— —	COMPLETED
NCT03469544	Long Non Coding RNA HOTAIR and Midkine as Biomarkers in Thyroid Cancer	HOTAIR	Thyroid Cancer	Diagnostic biomarker	OTHER: complete blood picture,serum urea and creatinine,liver function test,T3,T4,thyroid stimulating hormone,thyroglobuline and thyroglobuline anti body specific test Real time polymerase chain reaction	UNKNOWN
NCT02304471	Circulating lncRNA and CV Morbidities in CKD and ESRD	— —	Chronic Kidney Disease|End-stage Renal Disease	Prognostic biomarkers	— —	UNKNOWN

In preclinical studies, lncRNAs have shown promise as valuable biomarkers for diagnosing and predicting the prognosis of different cancer types. For example, recent research has identified C5orf66 antisense RNA 1 as a diagnostic marker for early GC, with an area under the curve (AUC) value of 0.789 (169). Combining multiple lncRNAs often enhances biomarker performance. For instance, a combination of lncRNA PANDAR, FOXD2-AS1 and SMARCC2 increases the AUC value to 0.84, indicating improved diagnostic accuracy (170). Similarly, an 11-lncRNA signature has been proposed as a prognostic indicator for BC, independent of various clinicopathological parameters (171). While numerous studies await validation in clinical settings, the utilisation of lncRNA expression profiles in cancer holds promise for future applications in early detection and prognosis, offering high accuracy and specificity.

As lncRNAs exhibit dysregulated expression in cancer and can modulate key tumorigenic processes by promoting or inhibiting different RCDs, they hold potential as diagnostic and prognostic biomarkers. For instance, plasma-derived ZFAS1, SNHG11, LINC00909 and LINC00654 together demonstrated a strong diagnostic capability for CRC with an AUC of 0.937, particularly in early-stage disease (AUC: 0.935) (172). Additionally, a nine-pyroptosis-associated lncRNA signature has been independently validated as a prognostic indicator for patients with BC. The AUC of this lncRNA signature associated with pyroptosis reached 0.880 in the training dataset and 0.799 in the validation dataset (173). As their regulatory roles in non-apoptotic RCDs are gradually being unveiled, lncRNAs are emerging as effective diagnostic and prognosis biomarkers in diseases. For instance, a clinical trial reveals that lncRNA NBR2 regulates septic endothelial pyroptosis, thereby underscoring the prognostic significance of pyroptosis levels in patients with sepsis (NCT04427371). Furthermore, lncRNAs regulating non-apoptotic RCDs pathways hold promise as tumour diagnostic markers. Integrating specific lncRNAs with other biomarkers has the potential to enhance diagnostic sensitivity and specificity across various cancer types. In conclusion, the integration of lncRNAs with traditional biomarkers will enhance diagnostic sensitivity and specificity, particularly in specific diseases or cellular subtypes.

### 5.2 Therapeutic potential of lncRNAs

Recent studies have unveiled the capacity of lncRNAs to influence RCD processes in cancer cells, presenting a potential avenue for suppressing tumour development. Understanding the mechanisms underlying gene overexpression or knockdown could provide new insight for targeting lncRNAs in treatment strategies. Currently, lncRNA-focused therapeutic strategies primarily involve RNA interference (RNAi) and antisense oligonucleotides (ASOs), offering customisable options for targeting a diverse range of transcripts (174) (**Figure 4B**). Preclinical models of human tumours have demonstrated the potential of lncRNAs as targets for cancer therapy. For example, the targeting of lncRNAs (e.g. LINC00336) using shRNA has shown promise in reducing cancer cell growth by promoting ferroptosis, suggesting these lncRNAs may represent novel therapeutic targets for human cancer (19, 122). In other studies, silencing of lncRNAs MAYA, MALAT1 and lncARSR using ASO has been shown to attenuate metastasis in mouse models, underscoring the therapeutic potential of ASOs targeting lncRNAs in cancer (175–178). Despite the emergence of preclinical studies highlighting the therapeutic potential of lncRNAs in cancer, further clinical trials are warranted to elucidate the targeting of lncRNAs as novel therapeutic options in clinical settings. Several ongoing clinical trials focus on anticancer therapy targeting non-apoptotic RCDs processes in cancers such as GC and HCC (e.g., NCT05334849, NCT04767750). Additionally, targeting mediators of non-apoptotic RCDs holds promise as candidates to enhance the efficacy and safety of therapeutic interventions. For instance, clinical trials evaluating the potential efficacy of targeting necroptosis in metastatic solid cancer (NCT04739618) and assessing the clinical efficacy of Aurora kinase A (AURKA), a negative regulator of necrosome activation, inhibitors in pancreatic ductal adenocarcinoma (NCT04479306, NCT04555837, NCT04085315 and NCT01924260) are underway. However, the clinical application of targeting lncRNAs is still in its early stages, necessitating extensive research to develop advanced strategies and effective agents for clinical settings. Furthermore, emerging studies have revealed that targeting specific lncRNAs (e.g. H19) is associated with various types of cell death in human cells, suggesting that targeting these lncRNAs could be a promising strategy for cancer therapy (123, 179). However, the precise mechanism by which lncRNAs facilitate or impede various RCD processes in cancer cells by targeting shared key molecules remains to be elucidated tumour prognosis by initiating multiple RCD mechanisms, offering novel insights for both diagnosis and treatment strategies.

Nonetheless, the current research on the role of lncRNAs as biomarkers or therapeutic targets has some specific limitations that require further study in cancer therapy. Although non-apoptotic RCD is a significant programmed cell death process that moderates the inflammatory response and cell demise in cancer, the investigation into how lncRNAs affect cancer therapy by modulating non-apoptotic RCD has not been thoroughly investigated in clinical trials, and this represents a potential area of exploration.

## 6 Conclusion and perspectives

Recent studies have highlighted the association between dysregulated expression of lncRNAs and the onset and progression of various cancers. Dysregulation of non-apoptotic RCDs, including necroptosis, pyroptosis and ferroptosis, has been shown to play a significant role in the etiopathogenesis of cancer (180). This review provides an overview of the functions and mechanisms of lncRNA-mediated ferroptosis, pyroptosis and necroptosis in tumour initiation and progression, emphasising the diagnostic, prognostic and therapeutic implications of lncRNAs in cancers. Thus, gaining a comprehensive understanding of the correlation between lncRNAs and non-apoptotic RCDs could offer valuable insights into the underlying molecular mechanisms of cancer pathogenesis. Moreover, inducing cancer cell death by targeting theses lncRNAs presents a promising strategy for cancer therapy.

However, several critical questions remain unanswered. Firstly, how can lncRNAs be used as reliable biomarkers for cancer diagnosis or prognosis, and how can they be explored further as therapeutic targets? While lncRNAs have shown promise as biomarkers and therapeutic targets, further research is needed to fully exploit their potential in clinical applications (181). A large quantity of lncRNAs modulate non-apoptotic RCDs and related pathways in tumour cells to exert oncogenic or anticancer effects, indicating potential value as predictive biomarkers of cancer chemotherapy responsiveness and clinical prognosis (74, 182, 183). Secondly, it is crucial to understand how to specifically target cancer cells to promote non-apoptotic RCDs and overcome treatment resistance. It is also important to understand the common signalling pathways (e.g., caspase-8 and RIPK1) between non-apoptotic RCDs and apoptosis, which are critical for targeting non-apoptotic RCDs regulated by lncRNAs in cancer therapy (184). Thirdly, targeting non-apoptotic RCD associated lncRNAs to reshape the TME could accelerate the development of therapeutic strategies for cancer therapy. Recent researches have highlighted that abnormally expressed lncRNAs can contribute to the modulation of the TME via non-apoptotic RCDs (185–187). Currently, the integration of novel technologies, such as CRISPR screening and single-cell sequencing to accurately identify genetic perturbations in different components of the TME will aid in the development of lncRNA-based therapeutics for clinical practice.

To date, only a limited number of chemical drugs and herbal remedies have been identified to target lncRNAs in non-apoptotic RCDs for cancer treatment, primarily focusing on understanding the regulatory mechanisms of non-apoptotic RCDs. Therefore, the development of lncRNA-targeted anticancer strategy, particularly involving herbal remedies, holds significant promise and may contribute to a more comprehensive clinical approach to enhancing cancer therapeutic outcomes.
